# Chronic Inflammatory Demyelinating Polyneuropathy With Reversible Severe Cognitive Impairment and Gastrointestinal Dysfunction

**DOI:** 10.7759/cureus.49341

**Published:** 2023-11-24

**Authors:** Madison M Patrick, Rachel Bielling, Galen Postma, Brenda Trokthi, Charles G Maitland

**Affiliations:** 1 Clinical Sciences, Florida State University College of Medicine, Tallahassee, USA; 2 Clinical Research, Tallahassee Memorial HealthCare, Tallahassee, USA

**Keywords:** novel phenotypic variant, optic atrophy, peripheral neuropathy, reversible gastrointestinal dysfunction, reversible cognitive decline, chronic inflammatory demyelinating polyneuropathy

## Abstract

We treated a patient with an unusual case of reversible rapidly progressive cognitive impairment, gastrointestinal dysfunction, and generalized neuromyopathy in chronic inflammatory demyelinating polyneuropathy (CIDP) with optic neuropathy.

A man in his 50s presented with a four-month history of rapidly progressive cognitive decline in addition to a six-month history of proximal greater than distal painful muscle weakness, wasting in all extremities, almost complete loss of deep tendon reflexes in his lower extremities, and slow progressive vision loss. Additionally, he had a 90-pound weight loss over the past two years with loss of appetite and ongoing chronic diarrhea. The exam showed muscle weakness and wasting with absent deep tendon reflexes. Initial Saint Louis University Mental Status (SLUMS) exam score was 16/30. Visual acuity was 20/25 with full extraocular movements; optical coherence tomography revealed superior arcuate bundle thinning bilaterally. Gastrointestinal workup proved nonrevealing. Serologic studies for vitamin deficiencies, heavy metals, and autoantibodies were negative. Whipple, *Giardia lamblia*, and *Campylobacter jejuni* stool testing were negative. Imaging studies were unremarkable. Nerve conduction studies showed demyelinating sensorimotor peripheral neuropathy. Muscle biopsy was indicative of denervation with scattered myopathic changes; no evidence of inflammatory myopathy nor glycogen or mitochondrial abnormalities was seen.

Intravenous immunoglobulin treatment was begun. The patient was started at a dose of 0.75g/kg every three weeks. Following good but incomplete clinical improvement after the first treatment, his dose was increased to 1g/kg every three weeks. He improved remarkably after four months of infusions, scoring 30/30 on SLUMS with a full return of muscle strength and reflexes. Diarrhea remitted. Visual acuity and conduction delay remained unchanged.

Symptom timing and dramatic response to immunoglobulins suggest a common immunological mechanism. In light of extensive differential investigations, unremarkable imaging and serology, and no other systemic disease processes, this case plausibly represents a potential new CIDP phenotypic variant.

## Introduction

Chronic inflammatory demyelinating polyneuropathy (CIDP) is characterized by muscle weakness and impaired sensory function across a range of presentations [1]. Further challenging in diagnosing CIDP is the varied temporality of the syndrome; although normally following a progressive onset and course, acute, subacute, and relapsing-remitting presentations have been demonstrated [2]. Underlying these diverse manifestations of CIDP is the common presumed mechanism of chronic inflammatory neuropathies (CINs); autoantibody-mediated demyelination of peripheral nerves [3]. The heterogeneous nature of CIDP makes diagnosis of a specific subtype challenging. Fortunately, the preponderance of CIDP diagnoses can be made with electrodiagnostic evidence of peripheral nerve demyelination coupled with a good response to steroid therapy and intravenous immunoglobulins (IVIg) [1,4].

There is an incredible paucity of information regarding CIDP and cognitive decline. The few reports are largely anecdotal and unsupportive of a direct causal link between CIDP and cognitive impairment [5]. Aside from neurological manifestations, new studies have delved into a potential link between gastrointestinal dysfunction and CIDP [6,7]. Important to these investigations is identifying the precipitating event, i.e., if a gut antigen triggers an immune reaction to cause CIDP, or if the autoimmunity theory holds and an aberrant immune response brings about gastrointestinal symptoms.

Here, we present a highly unusual case of CIDP; synchronously displaying rapidly progressive cognitive decline, severe gastrointestinal dysfunction, and bilateral optic nerve atrophy amongst other neuromyopathic symptoms. An extensive differential was established and evaluated before diagnosing CIDP. Further supporting this singular pathology is the complete reversal of cognitive decline and gastrointestinal dysfunction, with a full return of muscle strength and reflexes following empiric IVIg treatment. This case represents a putative new phenotypic variant of CIDP.

## Case presentation

A man in his 50s presented with a four-month history of rapidly progressive cognitive decline in addition to a six-month history of gradually progressive proximal greater than distal muscle weakness, patient-described “bone” pain, generalized moderate wasting in all extremities, almost complete loss of deep tendon reflexes (DTRs) in his lower extremities, vision loss, severe daily headaches, dysarthria, and intermittent myoclonic jerks. Additionally, he had a 90-pound weight loss over the past two years with loss of appetite and ongoing chronic diarrhea. Past medical and surgical history includes a Heller myotomy five years prior and a single grand mal seizure treated with Keppra for six months one year before clinic presentation. One month before the examination, the patient was seen in the emergency department for complaints of dysarthria, word-finding difficulties, frequent falls, and worsening intermittent diplopia over the preceding two months. This prompted magnetic resonance imaging (MRI) of the brain and whole spine with and without contrast, magnetic resonance angiography (MRA) of the head and neck without contrast, and computed tomography angiography (CTA) of the head and neck. These were all unremarkable.

Serologic studies revealed an unremarkable complete blood count. There were no deficiencies of Vitamin B12, folate, magnesium, copper, or zinc. Blood urea nitrogen, alkaline phosphatase, C-reactive protein, and creatinine kinase were normal. The heavy metal profile of the patient’s urine showed a normal creatinine with a mild elevation in total arsenic (44 ug/L), which is commonly seen in individuals residing in his geographic area. Inorganic, MMA, DMA, and total toxic arsenic were within normal. Lead was not detected. Further testing was negative for human immunodeficiency virus (HIV) p24 antigen and HIV antibodies. An antineutrophilic cytoplasmic antibody (ANCA) panel consisting of anti-Ro (SS-A), anti-La (SS-B), anti-myeloperoxidase, antiproteinase 3, cytoplasmic-ANCA, perinuclear-ANCA, and atypical perinuclear-ANCA was negative. An extended Sjogren’s antibody profile, comprised of anti-Fodrin immunoglobulin G (IgG) and immunoglobulin A (IgA) with repeat anti-Ro and anti-La studies, was negative. Tropheryma whipplei serum polymerase chain reaction (PCR) testing was negative. Stool testing for *Giardia lamblia* and *Campylobacter jejuni* was negative.

**Figure 1:**
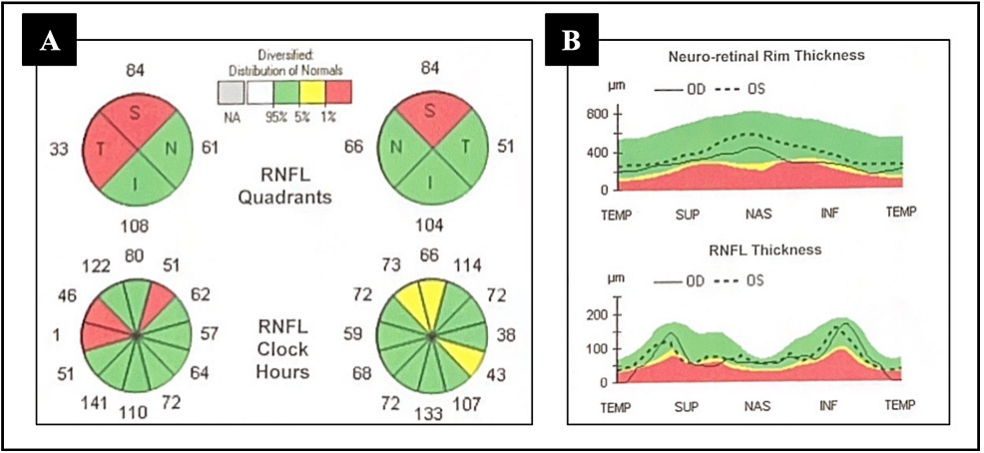
Optical coherence tomography (OCT) at initial presentation. OCT demonstrating superior arcuate bundle drop out (A) with decreased neuro-retinal rim thickness and decreased average retinal nerve fiber layer thickness (B) from initial patient presentation.

Initial mental status testing with the St. Louis University Mental Status (SLUMS) examination was 16/30, with scores under 21 categorized as dementia. Visual acuity was 20/25 bilaterally with full and intact extraocular muscles. Fundoscopic exam showed superior arcuate bundle dropout, decreased neuro-retinal rim (NRR) thickness, and decreased average retinal nerve fiber layer (RNFL) thickness; confirmed by optical coherence tomography (OCT) and depicted in Figures 1A, 1B. Visually evoked potential (VEP) demonstrated bilateral optic nerve conduction delay. Long bone x-rays were unremarkable. Electromyography (EMG) and nerve conduction studies (NCSs) of the right upper and right lower extremities revealed a length-dependent demyelinating sensorimotor peripheral neuropathy. Sensory nerve conduction, motor nerve conduction, and F-wave results from EMG and NCS are shown in Tables 1-3, respectively. As these results could not fully exclude the possibility of a superimposed myopathy, a muscle biopsy was performed. Biopsy of the left deltoid and left quadriceps demonstrated scattered small angulated atrophic myofibers with nuclear clumps; findings were indicative of denervation. There was no histopathologic evidence of inflammatory myopathy. Enzyme histochemistry of the samples did not suggest glycogen or mitochondrial abnormalities.

**Table 1:** Sensory nerve conduction studies of the right upper and lower limbs (RU/LL) showing absent or low amplitude RU/LL sensory responses. All testing was done at 37.6°C. When combined with the motor nerve conduction study (Table 2) and F-wave (Table 3) results, these findings demonstrate length-dependent demyelinating sensorimotor peripheral neuropathy. NR: none recorded. R: right. Ref: reference.

Sensory Nerve Conduction Study Results									
Nerve/Sites	Recording Site	Onset Latency (ms)	Peak Latency (ms)	Amplitude (µV)	Segments	Distance (cm)	Peak Difference (ms)	Velocity (m/s)	Area (µVms)
R Ulnar – Digit V (Antidromic)									
Wrist	Digit V	NR	NR	NR	Wrist – Digit V	11		NR	NR
Ref.			<3.1	>15.0	Ref.				
B. Elbow	Digit V	NR	NR	NR	B. Elbow – Wrist	25	NR	NR	NR
A. Elbow	Digit V	NR	NR	NR	A. Elbow – B. Elbow	10	NR	NR	NR
					A. Elbow – Wrist	35	NR	NR	
R Median – Digit II (Antidromic)									
Wrist	Digit II	NR	NR	NR	Wrist – Digit II	13		NR	NR
Ref.			<3.4	>20.0	Ref.				
R Radial – Anatomical Snuff Box (Forearm)									
Forearm	Wrist	NR	NR	NR	Forearm – Wrist	10		NR	NR
Ref.			<2.9	>15.0	Ref.				
R Sural – Ankle (Calf)									
Calf	Ankle	NR	NR	NR	Lateral Leg – Ankle	14		NR	NR
Ref.			<4.2	>5.0	Ref.				
R Superficial Peroneal – Ankle									
Lateral Leg	Ankle	NR	NR	NR	Lateral Leg - Ankle	14		NR	NR
Ref.			<4.2	>5.0	Ref.				

**Table 2:** Motor nerve conduction studies of the right upper and lower limbs (RU/LL) showing slowing of motor nerve conduction velocities with either absent or low amplitude RU/LL motor responses. All testing was done at 37.6°C. When combined with the sensory nerve conduction study (Table 1) and F-wave (Table 3) results, these findings demonstrate length-dependent demyelinating sensorimotor peripheral neuropathy. A. Elbow: Above elbow. ADM: abductor digiti minimi. AH: abductor hallucis. APB: abductor pollicis brevis. B. Elbow: Below elbow. EDB: extensor digitorum brevis. Fib head: fibular head. NR: none recorded. Pop fossa: popliteal fossa. R: right. Ref: reference

Motor Nerve Conduction Study Results									
Nerve/Sites	Muscle	Latency (ms)	Amplitude (mV)	Duration (ms)	Segments	Distance (cm)	Latency Difference (ms)	Velocity (m/s)	Area (mVms)
R Median – APB									
Wrist	APB	5.0	10.8	5.0	Wrist – APB	7			29.8
Ref.		<4.4	>4.0		Ref.				
Elbow	APB	10.3	8.6	4.9	Elbow – Wrist	22.5	5.3	43	21.5
Ref.					Ref.			>49	
R Ulnar – ADM									
Wrist	ADM	4.1	7.5	6.3	Wrist – ADM	7			28.6
Ref.		<3.6	>5.0		Ref.				
B. Elbow	ADM	9.1	6.6	6.9	B. Elbow – Wrist	25	5.0	50	27.3
Ref.					Ref.			>49	
A. Elbow	ADM	11.8	6.3	7.4	A. Elbow – B. Elbow	10	2.7	37	26.8
Ref.					Ref.			>49	
					A. Elbow – Wrist	35	7.7	37.6	
R Peroneal – EDB									
Ankle	EDB	5.3	3.4	6.2	Ankle – EDB	8			11.4
Ref.		<6.2	>2.0		Ref.				
Fib head	EDB	15.1	2.6	7.5	Fib head – Ankle	36	9.8	37	10.9
Ref.					Ref.			>39	
Pop fossa	EDB	17.3	2.4	7.5	Pop fossa – Fib head	8	2.1	37	9.3
Ref.					Ref.			>39	
					Pop fossa – Ankle	44	12.0	37	
R Tibial – AH									
Ankle	AH	5.4	1.9	8.9	Ankle – AH	8			8.9
Ref.		<6.0	>3.0		Ref.				
Pop fossa	AH	18.2	1.8	8.7	Pop fossa – Ankle	38	12.8	30	3.9
Ref.					Ref.			>39	

**Table 3:** F-wave results of the right upper and lower limbs (RU/LL) showing slowing of motor nerve conduction velocities with either absent or low amplitude RU/LL motor and sensory responses. All testing was done at 37.6°C. When combined with the sensory nerve conduction study (Table 1) and motor nerve conduction study (Table 2) results, these findings demonstrate length-dependent demyelinating sensorimotor peripheral neuropathy. ADM: abductor digiti minimi. AH: abductor hallucis. APB: abductor pollicis brevis. R: right. Ref: reference.

F Wave Results	
Nerve	F Latency (ms)
R Median – APB	32.0
Ref.	<32.0
R Ulnar – ADM	37.5
Ref.	<32.0
R Tibial – AH	65.5
Ref.	<58.0

Treatment with 0.75g/kg body weight IVIg every three weeks was begun. The patient improved following the completion of the first round of treatment. SLUMS increased to 23/30, falling into the mild cognitive impairment range. Muscle strength and gastrointestinal dysfunction improved, albeit mild generalized weakness was still apparent in his extremities, and continued but infrequent diarrhea. Given good but incomplete clinical improvement, his IVIg was increased to 1.0g/kg body weight every three weeks. Following four months of treatment, cognitive decline reversed completely, with the patient improving to a score of 30/30. Full muscle strength and DTRs returned in both upper and lower extremities. Gastrointestinal dysfunction remitted. Visual acuity held steady at 20/25. Repeat OCT demonstrated bilateral optic nerve fiber bundle dropout in a superior arcuate distribution; the visual field correlated with this. A summary of the patient’s initial presentation and improvement of symptoms after IVIg initiation is shown in Table 4. The patient continued to receive IVIg every three weeks for a year and followed up with the clinic every three months. He has since been able to return to his daily activities, including his exercise regimen, and is doing remarkably well.

**Table 4:** Clinical course from presentation to one- and four-month follow up. Initial patient symptoms with improvement demonstrated at one- and four-month follow ups after starting immunoglobulin treatment. Cognition was quantified by St. Louis Mental Status examination. DTRs: deep tendon reflexes. NRR: neuro-retinal rim. OCT: optic coherence tomography. RNFL: retinal nerve fiber layer. VEP: visual evoked potential.

Category	Initial Presentation	1 Month Follow Up	4 Month Follow Up
Musculoskeletal	Global extremity wasting; severe proximal > distal extremity weakness	Wasting improved but below baseline; mild continued proximal > distal weakness	Near baseline muscle bulk; weakness fully resolved
Reflexes	Bilateral Achilles and left patellar reflexes absent; right patellar reflex 1+; bilateral biceps and triceps reflexes 2+	Full return of DTRs	Full return of DTRs
Vision	Acuity: 20/25 bilaterally. OCT: Bilateral superior arcuate bundle drop out; bilateral decreased NRR and RNFL thickness. VEP: Bilateral optic nerve conduction delay.	Unchanged	Unchanged
Cognition	Dementia (16/30)	Mild cognitive impairment (23/30)	Normal cognition (30/30)
Gastrointestinal	Daily diarrhea; appetite loss	Infrequent diarrhea; full appetite	Resolved diarrhea; full appetite

## Discussion

Differential diagnosis 

An extensive differential diagnosis was created. The initial diagnosis of inclusion body myopathy (IBM) associated with Paget disease of bone (PDB) and frontotemporal dementia (FTD) (IBMPFD) was made with the patient’s self-described bone and muscle pain, muscle weakness, optic neuropathy, and dementia. IBMPFD is characterized by progressive muscle weakness involving proximal and distal muscles, focal areas of bone pain and potential pathological fractures, various cognitive impairments, and vision impairment from optic canal compression [8]. Long bone imaging was performed to evaluate for PDB with NCS and muscle biopsy planned for IBM. Following unremarkable bone imaging and muscle biopsy, IBMPFD was ruled out. Given the NCS results, etiologies of demyelinating polyneuropathy were explored. 

Systemic diseases that precipitate immune-mediated neuropathies were ruled out, including metachromatic leukodystrophy (MLD) and mitochondrial neurogastrointestinal encephalomyopathy (MNGIE). Clinically, adult-onset MLD presents with intellectual and behavioral changes followed by polyneuropathy and potential visceral organ involvement [9]. Linear hypointensities within hyperintense regions of demyelinated white matter in periventricular regions create the pathognomonic “stripe” or “leopard skin” sign in MLD [10]. MNGIE is a relentlessly progressive and degenerative disease due to a *TYMP* gene mutation impairing thymidine phosphorylase [11]. Clinical findings include gastrointestinal dysfunction, ocular manifestations, nervous system impairments; characteristically peripheral neuropathy, and absent reflexes [12]. MRI will demonstrate leukoencephalopathy, and diagnosis is made with serum and urine studies or gene sequencing [12]. In the setting of unremarkable imaging studies, no further genetic testing was performed for either MLD or MNGIE.

Paraneoplastic neuropathies (PNs), such as sensorimotor neuropathy (SMN) and autonomic neuropathy (AN), were also considered. SMN is the most common yet enigmatic PNs with no pathogenomic characteristics identified. However, neurologic impairment does not occur in SMN, which is a focal aspect of our patient’s syndrome [13]. AN was investigated as a cause of gastrointestinal dysmotility. It is typically seen in amyloid light (AL) chain amyloidosis in association with other paraneoplastic neurological syndromes (PNS), such as Lambert-Eaton myasthenic syndrome [13]. Considering myopathy is seen in only 1.5% of AL amyloidosis cases and lack of heart or kidney involvement in this patient, amyloidosis is an unlikely diagnosis for this case [14]. Ultimately, NCS demonstrating demyelinating polyneuropathy, the wide variety of CIDP phenotypes, and the dramatic response to empiric IVIg therapy make CIDP a much more likely diagnosis in this patient.

Chronic inflammatory demyelinating polyneuropathy 

CIDP is characterized by slowly progressive sensorimotor involvement developing over at least two months. An aberrant immune response targets the peripheral nervous system, leading to demyelination and secondary axonal injury. There are a number of phenotypic variants; among those, typical CIDP, Lewis-Sumner syndrome, and focal CIDP involve sensory and motor systems as is seen here [15]. Typical CIDP describes proximal and distal musculature involvement at onset that progresses in a symmetrical fashion. Our patient presented with symmetric, severe demyelinating polyneuropathy preceding a litany of symptoms extending beyond the well-described CIDP variants, providing support for a potential novel phenotypic variant of CIDP.

Mental status decline in CIDP patients is usually considered ancillary to the primary disease with no causal link established between the two [5]. Newer investigations into cognition in CIDP have demonstrated various mild neurofunctional impairments, specifically with information processing speed, flexibility, and verbal processing. This study further suggested a potential mechanism of blood-brain barrier dysfunction allowing extravasation of autoantibodies into the central nervous system underlying the impairments [3]. While the functional decline has been demonstrated, the rapidly progressive mental status decline is wholly unexpected in CIDP, with only one previous report identifying this. Dorman and Samaniego described a CIDP patient with unremarkable imaging studies presenting with a rapidly progressive dementia defined by a 12/30 on the initial Montreal Cognitive Assessment (MoCA). Empiric IVIg for CIDP was initiated; following three weeks of treatment the patient’s cognitive function returned, demonstrated by a MoCA of 29/30 [16]. Similarly, our patient showed severe cognitive decline reversed with IVIg treatment, implicating an immunological mechanism.

Gastrointestinal dysfunction in autoimmune neuropathies and dysautonomias has a wide variety of presentations. Upper tract symptoms, such as dysphagia, achalasia, and gastroparesis, can be coupled with lower tract involvement of diarrhea, fecal incontinence, and constipation, among others [17]. Gastrointestinal involvement in CIDP specifically is rare, even among a host of uncommon autonomic dysfunctions [7]. The disease course can become unclear, with debate over which syndrome is the etiology versus the manifestation. Studies have discussed a potential gut antigen precipitating a humoral and cellular response that triggers CIDP onset [6]. However, the more commonly propagated autoimmunity theory of an aberrant immune response could likewise precipitate gastrointestinal symptoms. Although the absence of conclusive gastrointestinal studies supersedes a definitive diagnosis, the reversal of gastrointestinal symptoms and CIDP simultaneously with IVIg further support a common etiology underlying both syndromes.

## Conclusions

To date, no cases have been reported of CIDP presenting with reversible cognitive impairment and gastrointestinal dysfunction. The symptom onset, timing, progression, and dramatic response to immunoglobulin therapy suggest a common immunological mechanism underlying these morbidities. Limitations of this article would include the nature of all case reports, which being a single novel presentation cannot be immediately generalized to the public. However, given the few reports outlining cognitive decline in CIDP, in addition to the studies investigating links between CIDP and GI dysfunction, there is evidence that these manifestations of CIDP can occur individually. In light of extensive differential investigations, unremarkable imaging, serologic studies, and no other systemic disease processes, this case can plausibly represent a potential new phenotypic variant of CIDP.
